# On the Use of Reflection Polarized Optical Microscopy for Rapid Comparison of Crystallinity and Phase Segregation of P3HT:PCBM Thin Films

**DOI:** 10.1002/marc.202400577

**Published:** 2024-10-21

**Authors:** Rawan A. Alzahrani, Nisreen Alshehri, Alaa A. Alessa, Doha A. Amer, Oleksandr Matiash, Catherine S. P. De Castro, Shahidul Alam, José P. Jurado, Julien Gorenflot, Frédéric Laquai, Christopher E. Petoukhoff

**Affiliations:** ^1^ Physical Sciences and Engineering Division (PSE) KAUST Solar Center (KSC) King Abdullah University of Science and Technology (KAUST) Thuwal 23955‐6900 Kingdom of Saudi Arabia; ^2^ Physics and Astronomy Department College of Sciences King Saud University Riyadh 12372 Kingdom of Saudi Arabia; ^3^ Present address: Physical Chemistry and Spectroscopy of Energy Materials Department of Chemistry Ludwig‐Maximilians‐Universität München Butenandtstraße 5‐13 (E) D‐81377 München Germany

**Keywords:** crystallinity, microstructure, morphology, organic semiconductors, polarized light

## Abstract

Rapid, nondestructive characterization techniques for evaluating the degree of crystallinity and phase segregation of organic semiconductor blend thin films are highly desired for in‐line, automated optoelectronic device fabrication facilities. Here, it is demonstrated that reflection polarized optical microscopy (POM), a simple technique capable of imaging local anisotropy of materials, is capable of determining the relative degree of crystallinity and phase segregation of thin films of polymer:fullerene blends. While previous works on POM of organic semiconductors have largely employed the transmission geometry, it is demonstrated that reflection POM provides 3× greater contrast. The optimal configuration is described to maximize contrast from POM images of polymer:fullerene films, which requires Köhler illumination and slightly uncrossed polarizers, with an uncrossing angle of ±3°. It is quantitatively demonstrated that contrast in POM images directly correlates with 1) the degree of polymer crystallinity and 2) the degree of phase segregation between polymer and fullerene domains. The origin of the bright and dark domains in POM is identified as arising from symmetry‐broken liquid crystalline phases (i.e., dark conglomerates), and it is proven that they have no correlation with surface topography. The use of reflection POM as a rapid diagnostic tool for automated device fabrication facilities is discussed.

## Graphical abstract

The use of reflection polarized optical microscopy (POM) for imaging changes in crystallinity and phase segregation of (poly(3‐hexylthiophene‐2,5‐diyl)):([6,6]‐phenyl‐C_61_‐butyric acid methyl ester) (P3HT:PCBM) thin films is demonstrated. Methods to reduce aberrations, including the use of proper Köhler illumination, are discussed and demonstrated to maximize contrast. Bright and dark domains in POM images of P3HT:PCBM are identified as originating from dark conglomerate liquid crystalline states.

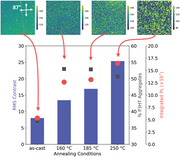

## 1 Introduction

Organic semiconductors are a unique class of materials for electronic and optoelectronic devices due to their large absorption cross‐sections,^[^ ^1^, ^2^ ^]^ synthetic tunability,^[^ ^3^ ^]^ and mechanical flexibility. As such, they have been employed as the active layers in organic solar cells (OSCs), light‐emitting diodes, thin film transistors, and thermoelectrics, particularly with the potential for cost‐effective, flexible, lightweight, or semi‐transparent devices.^[^ ^4^, ^5^, ^6^, ^7^ ^]^ Many organic semiconductors can be processed into thin films by casting from solution, which is one of the least energy‐intensive methods to prepare thin films. However, when casting from solution, organic semiconductors, particularly conjugated polymers, tend to form amorphous or semi‐crystalline films, which generally have low charge carrier mobilities. Crystallinity, as well as molecular orientation and morphology, can be controlled through different approaches, such as thermal annealing,^[^ ^8^, ^9^, ^10^ ^]^ additive engineering,^[^ ^11^ ^]^ polymer molecular weight,^[^ ^12^, ^13^, ^14^ ^]^ or choice of solvent.^[^ ^15^ ^]^ Precise control of the crystallinity, molecular orientation, and morphology of organic semiconductors is pivotal for achieving elevated charge carrier mobilities, which is directly related to device performance.^[^ ^8^, ^9^, ^10^, ^14^, ^15^, ^16^, ^17^ ^]^ An additional factor arises for OSCs, whose active layers generally consist of blends between electron donor and acceptor materials, which is control of the domain size and phase segregation between the blended materials.

A prototypical example of a blend that was used for OSC active layers for over a decade is based on the conjugated polymer donor, P3HT (poly(3‐hexylthiophene‐2,5‐diyl)) blended with the fullerene acceptor, PCBM ([6,6]‐phenyl‐C_61_‐butyric acid methyl ester).^[^ ^18^ ^]^ Despite the advances in OSC materials and device efficiencies over the past decade, P3HT:PCBM has still remained a model system for investigating novel characterization methods.^[^ ^19^, ^20^, ^21^, ^22^ ^]^ The crystallinity and morphology of P3HT:PCBM films have been well understood through a combination of characterization techniques, including scanning electron microscopy (SEM), transmission electron microscopy (TEM),^[^ ^9^, ^10^, ^23^ ^]^ atomic force microscopy (AFM),^[^ ^8^, ^16^ ^]^ electron tomography,^[^ ^10^ ^]^ grazing‐incidence X‐ray diffraction (GIXD) or scattering (GIWAXS/GISAXS),^[^ ^8^, ^23^, ^24^, ^25^, ^26^ ^]^ and small‐angle neutron scattering (SANS).^[^ ^17^ ^]^

While each of these techniques can give precise, quantitative information at nanometer or molecular length scales, they are either destructive (e.g., SEM, TEM, GIWAXS/GISAXS, SANS), time‐consuming (e.g., AFM), or potentially require access to a beamline facility (GIWAXS/GISAXS, SANS). None are particularly suitable for rapid, non‐destructive evaluation of the crystallinity and morphology of organic semiconductor thin films. In contrast, optical microscopy is a very simple, inexpensive, and non‐destructive tool that can be used to rapidly diagnose the quality of organic thin films.^[^ ^27^ ^]^ While typical optical microscopy based on bright‐field (BF) imaging provides very limited information about crystallinity or morphology, polarized optical microscopy (POM) can provide enhanced contrast for optically anisotropic materials, such as organic semiconductors.^[^ ^28^, ^29^, ^30^ ^]^ POM employs a set of orthogonal polarizers in the illumination and collection paths of the microscope. As such, only light that had a change in polarization upon transmission or reflection will be imaged. POM has been employed to image the anisotropy of organic semiconductors with a particular focus on imaging highly crystalline,^[^ ^31^, ^32^ ^]^ highly oriented,^[^ ^33^, ^34^, ^35^, ^36^ ^]^ or nanostructured^[^ ^37^, ^38^ ^]^ organic molecules (Table S1, Supporting Information). Most of these studies either used POM to identify the orientation of highly oriented polymer chains, or used imaging conditions which lacked the resolution or contrast required to image sub‐micron changes in the contrast of spin‐coated organic films typical for optoelectronic devices. The very few studies that have utilized POM as a diagnostic tool for spin‐coated organic films with preparation conditions typical for optoelectronic device fabrication^[^ ^19^, ^39^, ^40^, ^41^ ^]^ have been qualitative in nature ‐ lacking any quantitative metrics to compare between samples with different degrees of crystallinity.

**Figure 1:**
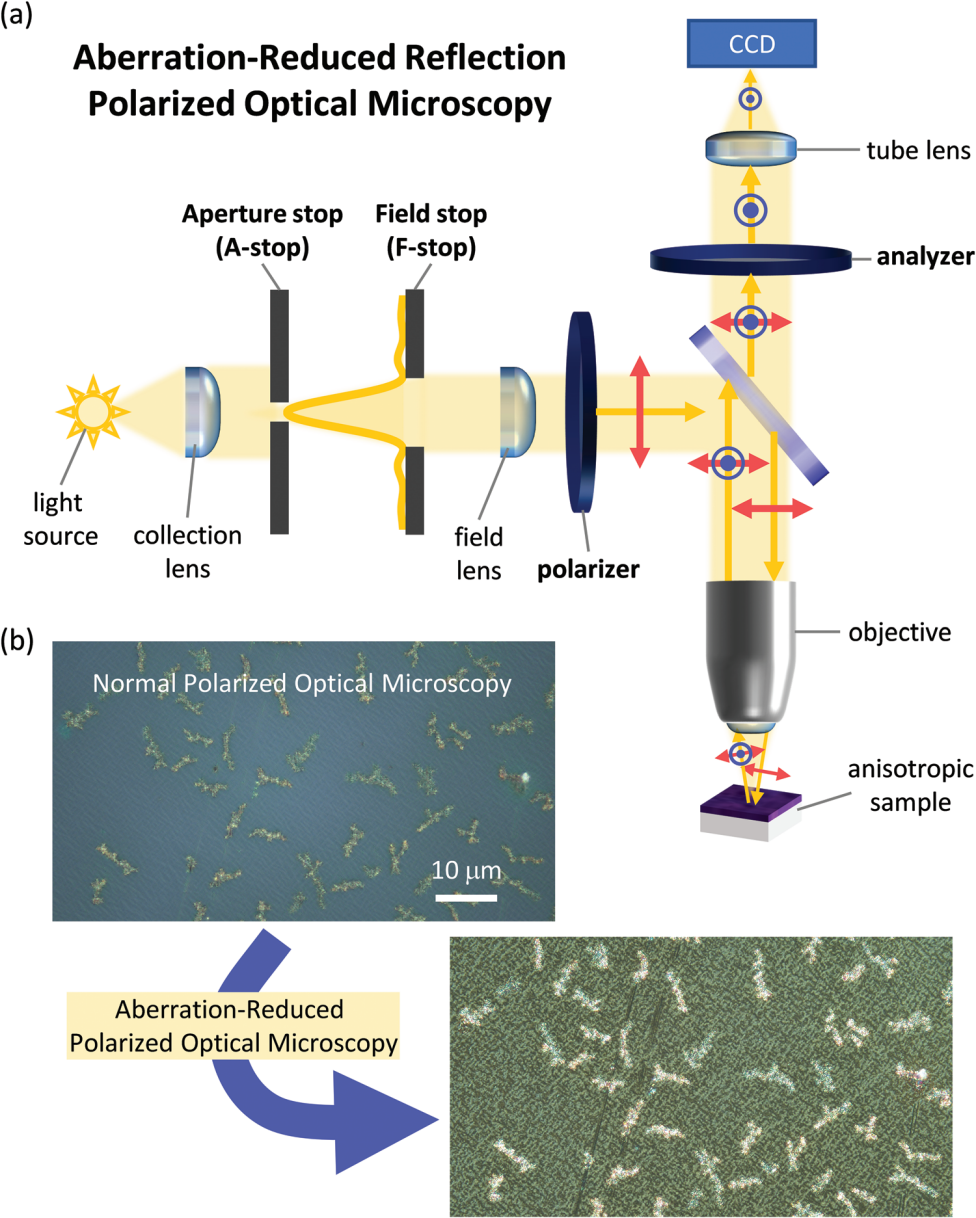
a) Schematic of aberration‐reduced reflection polarized optical microscope (POM) experiment using Köhler illumination. The yellow arrows represent the direction of light propagation; the red arrows represent the incident polarization direction; the blue circled dots represent the rotated polarization in the out‐of‐plane direction. b) Example images of POM from P3HT:PCBM films annealed at 185 °C acquired: 1) without proper Köhler illumination, where the A‐ and F‐stops were fully opened, leading to significant aberrations, and 2) with proper Köhler illumination, where the A‐ and F‐stops have been closed as described in the Supporting Information. The images are from identical regions and were acquired with a polarizer‐analyzer uncrossing angle of 3° (i.e., 87° between the polarizer and analyzer) with a 50× objective lens.

In this work, we employed POM in the reflection geometry, which provides greater sensitivity to changes in polarization compared with the widely used transmission geometry. Through the use of Köhler illumination and control of the microscope's aperture and field stops, we reduced the aberrations typically present in many POM images of organic semiconductor thin films previously reported (**Figure** **1**). We demonstrate the optimal configuration of a polarizing optical microscope experiment to capture the sub‐micron changes in the local degree of crystallinity within typical spin‐coated P3HT:PCBM films. We quantitatively compare different optical microscopy imaging conditions that led to the most pronounced contrast, including BF, POM with crossed polarizers, and POM with slightly uncrossed polarizers, in both reflection and transmission geometries. We show that the maximum contrast enhancement was achieved for reflection POM acquired with slightly uncrossed polarizers, having an uncrossing angle of ±3°. Using our optimized imaging conditions, we demonstrate that the contrast in POM directly correlates with the degree of P3HT crystallinity within P3HT:PCBM films. We further show that the contrast in POM increases even further for high degrees of phase segregation between P3HT and PCBM. We show that the bright and dark domains observed in POM exhibit optical activity and deduce that they likely originate from symmetry‐broken liquid crystalline phases, having no correlation with surface topography. Finally, we discuss the potential use of reflection POM as a rapid diagnostic tool for in‐line fabrication of organic optoelectronic devices.

## 2 Results and Discussion

### 2.1 Reflection versus Transmission Polarized Optical Microscopy (POM)

In general, reflection is more sensitive to changes in polarization state than transmission, particularly at non‐normal angles of incidence.^[^ ^42^ ^]^ In optical microscopy, since light illuminates the sample through a microscope objective, all angles that fall within the objective's numerical aperture (NA) illuminate the sample. For an objective with NA of 0.8, all angles between 0° and ≈53° are incident on the sample. For tungsten‐halogen lamp illumination and imaging with a standard charge‐coupled device (CCD) camera, the central wavelength for optical microscopy is generally ≈550 nm to 650 nm. At this wavelength range, most of the widely employed organic semiconductors for visible optoelectronic applications have strong absorption coefficients, resulting in their Fresnel transmission coefficients being smaller than their reflection coefficients. Furthermore, the reflection geometry enables imaging from opaque samples, such as very thick organic films, or thin organic films deposited on opaque substrates.

In order to achieve the highest quality reflection POM images, it is necessary to employ Köhler illumination.^[^ ^43^, ^44^ ^]^ In reflected optical microscopy using Köhler illumination, a uniform illumination of the back focal plane of the objective must be achieved to create the most uniform illumination conditions of the sample, regardless of non‐uniformities in the light source. When Köhler illumination is not used, reflection POM images suffer from severe aberrations, such as spherical and coma aberrations, resulting in bright domains appearing smeared (Figure 1b and Figure S1, Supporting Information). The aperture and field stops of the microscope (Figure 1a) are pivotal to achieving proper Köhler illumination, to minimizing aberrations, and to maximizing imaging contrast (Figure 1b and Figure S1, Supporting Information).

**Figure 2:**
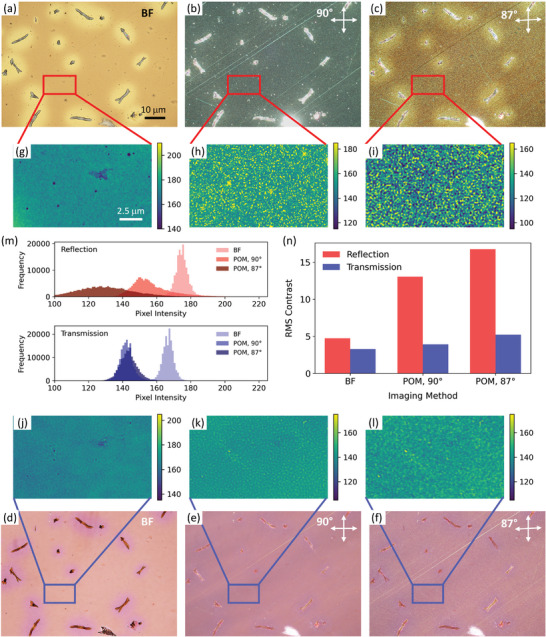
Comparison of different optical microscopy imaging conditions for maximizing contrast from a P3HT:PCBM film annealed at 185 °C. a–f) True‐color optical microscopy images acquired from an identical region, acquired using different configurations: a–c) reflection geometry; d–f) transmission geometry; a,d) bright‐field (BF); b,e) POM using crossed polarizers; c,f) POM using slightly uncrossed polarizers. All images were acquired using a 50× objective lens and share a common scale bar shown in (a). g–l) Grayscale regions of interest cropped from identical spots from the true‐color images. The grayscale images have been plotted on false color maps to visualize changes in contrast between samples. All images have a constant range of color intensities (±35 from their peak position) and share a common scale bar shown in (g). m) Histograms of the pixel intensity distribution for each imaging configuration, acquired from the grayscale regions of interest (g–l). n) Root‐mean‐square (RMS) contrast calculated for each imaging configuration, acquired from the grayscale regions of interest (g–l).

To demonstrate the greater sensitivity to changes in polarization by reflection compared to transmission, we compared several optical microscopy imaging conditions using both geometries (**Figure** **2**). Qualitatively, in reflection, BF images of P3HT:PCBM films annealed at 185 °C showed visible PCBM crystallites over a mostly uniform, bright background (Figure 2a). The POM image of the same region with crossed polarizers showed mostly bright PCBM crystals, due to their high crystallinity, with a relatively dark, yet textured background (Figure 2b). The background showed alternating dark and bright regions, which suggests local sub‐micron domains of varying crystallinity. When POM images were acquired using slightly uncrossed polarizers, with an uncrossing angle of 3° (i.e., 87° between the polarizer and the analyzer), the background contrast increased relative to the cross polarized geometry (Figure 2c). We note that the uncrossing angle of 3° gave the highest contrast compared to all other uncrossing angles (Figure S1d, Supporting Information). In the transmission geometry, there were not many changes that could be observed between BF, POM with crossed polarizers, and POM with slightly uncrossed polarizers (Figure 2d–f), apart from the intensity of the PCBM crystals and a slight increase in the apparent contrast of the background in POM.

To quantify the differences in contrast between different imaging conditions, we selected a 500 pixel × 300 pixel (i.e., 15 µm × 9 µm) region of interest from each image. These regions of interest were converted to grayscale and plotted on false color maps with a constant range of their color intensities (±35 from their peak position) (Figure 2g–l). From these grayscale images, visually, the reflection geometry had higher contrast than the transmission geometry for each imaging condition. The most texture in the image came from the reflection POM images in both cross polarized (Figure 2h) and slightly uncrossed polarized (Figure 2i) conditions. This was even more apparent by comparing the pixel histograms of each different imaging condition (Figure 2m), where the reflection geometries always had broader intensity distributions than the respective transmission geometries. Finally, the root‐mean‐square (RMS) contrast was quantified by calculating the deviation of pixel intensities from the mean intensity (Figure 2n). In the reflection geometry, the contrast increased from 4.7 for BF to 13.0 for POM with crossed polarizers to its maximum value of 16.8 for POM with slightly uncrossed polarizers. Meanwhile, in the transmission geometry, the contrast was considerably lower: 3.3 for BF; 3.9 for POM with crossed polarizers; and 5.2 for POM with slightly uncrossed polarizers. This confirms that POM in the reflection geometry is more sensitive to changes in polarization than POM in the transmission geometry.

### 2.2 Reflection POM Traces Changes in Crystallinity

**Figure 3:**
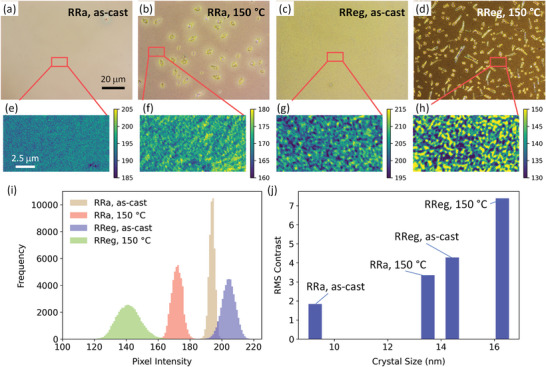
Reflection POM images of P3HT:PCBM films of varying degrees of crystallinity. Films were formed from either regio‐random (RRa) or regio‐regular (RReg) P3HT, each either as‐cast, or annealed at 150 °C. a–d) True‐color POM images of each P3HT:PCBM film, acquired using slightly uncrossed polarizers (3° uncrossing angle) and a 50× objective lens (common scale bar shown in (a)). Note, these images were published previously in our prior work,^[^ ^19^ ^]^ but are reproduced here to show the regions of interest for subsequent quantitative analysis. e–h) Grayscale regions of interest cropped from the red boxed regions in the true‐color images. The grayscale images have been plotted on false color maps to visualize changes in contrast between samples. All images have a constant range of color intensities (±10 from their peak position) and share a common scale bar shown in (e). i) Histograms of the pixel intensity distribution for each P3HT:PCBM film, acquired from the grayscale regions of interest (e–h). j) Root‐mean‐square (RMS) contrast calculated for each P3HT:PCBM film, acquired from the grayscale regions of interest (e–h). The crystal sizes were taken from the values reported in our prior work,^[^ ^19^ ^]^ which were determined from the Scherrer equation from XRD measurements.

Having demonstrated that aberration‐reduced reflection POM with 3° slightly uncrossed polarizers gives the maximum contrast for P3HT:PCBM thin films, we apply these imaging conditions to compare films with different degrees of P3HT crystallinity. We previously reported reflection POM images of P3HT:PCBM films with different degrees of crystallinity through the use of P3HT with different degrees of regioregularity (i.e., regio‐random, RRa and regio‐regular, RReg), with and without thermal annealing (reproduced in **Figure** **3**a–d).^[^ ^19^ ^]^ As we described previously, there was a qualitative increase in POM contrast in the mixed P3HT:PCBM regions with increasing regio‐regularity, and with thermal annealing. We now conducted a more quantitative analysis on these different POM images. We selected 300 pixel × 160 pixel (i.e., 13.8 µm × 7.4 µm) regions of interest from each image, which were then converted to grayscale and plotted on false color maps with a constant range of their color intensities (±20 from their peak position) (Figure 3e–h). From these grayscale images, and their resulting histograms (Figure 3i), the contrast of the POM images clearly increased both with increasing regio‐regularity and with thermal annealing. In fact, the RMS contrast increased with increasing P3HT crystal size (Figure 3j). The RRa films had the lowest crystal sizes, resulting in having significantly lower RMS contrast (1.84 for as‐cast and 3.35 for 150 °C annealed) compared to the RReg films (4.28 for as‐cast and 7.38 for 150 °C annealed). The most crystalline film, RReg, annealed, had over 4 times higher contrast than the least crystalline film (RRa, as‐cast). This demonstrates that the contrast observed in reflection POM is directly related to the degree of crystallinity of these semi‐crystalline organic thin films.

### 2.3 Reflection POM Traces Changes in Phase Segregation

**Figure 4:**
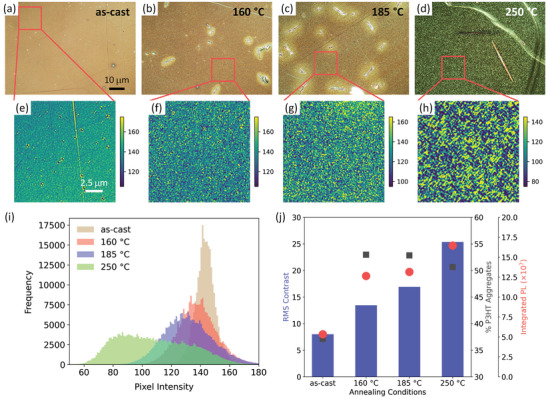
Reflection POM images of P3HT:PCBM films prepared with different thermal annealing conditions. a–d) True‐color POM images of each P3HT:PCBM film, acquired using slightly uncrossed polarizers (3° uncrossing angle) and a 50× objective lens (common scale bar shown in (a)). e–h) Grayscale regions of interest cropped from the red boxed regions in the true‐color images. The grayscale images have been plotted on false color maps to visualize changes in contrast between samples. All images have a constant range of color intensities (±35 from their peak position) and share a common scale bar shown in (e). i) Histograms of the pixel intensity distribution for each P3HT:PCBM film, acquired from the grayscale regions of interest (e–h). j) Root‐mean‐square (RMS) contrast calculated for each P3HT:PCBM film, acquired from the grayscale regions of interest (e–h). The % P3HT aggregates and integrated photoluminescence (PL) values, as reported in Figures S2 and S3 and Table S2 (Supporting Information) are overlaid.

Having compared reflection POM images from P3HT:PCBM films with well‐controlled changes in the degree of P3HT crystallinity, we next investigated the impact of thermal annealing temperature on the POM contrast for regio‐regular P3HT:PCBM films (**Figure** **4**). Here, we compared both the changes in the degree of P3HT crystallinity as well as the degree of phase segregation as we changed the thermal annealing conditions. The degree of P3HT crystallinity was monitored by spectral deconvolution of the absorption spectra using the Spano model^[^ ^22^, ^45^, ^46^ ^]^ (Figure S2 and Table S2, Supporting Information), and the degree of phase segregation was monitored by comparing the integrated photoluminescence (PL) spectra^[^ ^10^, ^47^, ^48^, ^49^ ^]^ (Figure S3 and Table S2, Supporting Information). Comparing the as‐cast film to the film annealed at 160 °C, the degree of P3HT crystallinity increased from 37.1% to 53.0%, and the integrated PL increased from 5.3 × 10^7^ to 12.7 × 10^7^ (Figures S2 and S3 and Table S2, Supporting Information). These effects led to an increase in the RMS contrast of reflection POM images from 8.0 to 13.4 (Figure 4a,b,e,f,j). Similar as shown in Figure 3, we attribute this contrast enhancement predominantly to changes in the degree of P3HT crystallinity. Although the integrated PL increased by a factor of 2.4, the domain sizes likely increased from only ≈10 nm for well‐inter‐mixed phases expected from as‐cast films^[^ ^48^ ^]^ to ≈25 nm at this annealing temperature^[^ ^50^ ^]^ ‐ sizes well‐below the diffraction limit of optical microscopy (≈400 nm).

As the annealing temperature increased to 185 °C, the RMS contrast increased to 16.9, despite the degree of P3HT crystallinity remaining roughly the same (52.8%; Figure 4c,g,j). The integrated PL increased to 13.2 × 10^7^, suggesting the degree of phase segregation continued to increase. As the annealing temperature reached 250 °C, there was a pronounced increase in the contrast of the reflection POM image, as evidenced directly in the true‐color image (Figure 4d), the grayscale region of interest (Figure 4h), the width of the histogram (Figure 4i), and the calculated RMS contrast (Figure 4j). The RMS contrast increased to 25.3 ‐ a nearly 50% increase from the film annealed at 185 °C, despite the degree of P3HT crystallinity dropping to 50.7%. Given that the integrated PL increased to 16.5 × 10^7^, the degree of phase segregation increased by ≈25%. However, the PCBM domain sizes were likely significantly larger at this temperature. PCBM‐rich domains (besides the micron scale, needle‐like crystallites) have been reported as large as several hundreds of nm.^[^ ^51^ ^]^ Additionally, 250 °C is in the optimal temperature range for promoting PCBM crystallization: it's larger than the melting temperature of P3HT (≈200 °C), less than the melting temperature of PCBM in 50:50 blends (≈275 °C), and larger than the melt crystallization temperature of pure PCBM (231.8 °C).^[^ ^52^ ^]^ This enhanced degree of phase segregation is further supported by the bimodal distribution of pixel intensities in the histogram for the 250 °C film (Figure 4i) ‐ a feature not observed for any of the other samples (Figures 4i and 3i).

Images taken in reflection BF and POM with cross polarized geometries for each of these samples are provided in Figure S4 (Supporting Information). Higher magnification images of the films with the highest contrast, using different imaging conditions, are provided in Figure S5 (Supporting Information).

### 2.4 Nature of the Bright and Dark Domains Observed in POM

To determine the nature of the intensity variations giving rise to enhanced contrast in POM images of P3HT:PCBM films, we correlated AFM surface topography measurements with identical regions imaged using POM for films annealed at 160 °C (Figure S6, Supporting Information). The details of the correlation analysis are provided in Figures S7 and S8 (Supporting Information). Conducting a pixel‐by‐pixel correlation showed that there was no correlation between a high‐resolution AFM scan and POM image taken at an identical region (Figure S6f, Supporting Information). This lack of correlation was confirmed with 4 separate regions, none of which showed a correlation between the surface topography and the intensity variations imaged using POM (Figure S9, Supporting Information), likely due to the low (<3.5 nm) RMS surface roughness of the P3HT:PCBM films (Figure S10, Supporting Information).

**Figure 5:**
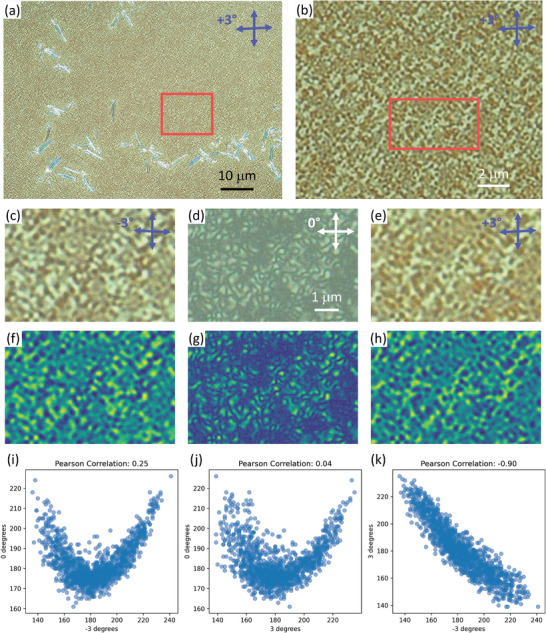
Optical activity of sub‐micron domains within P3HT:PCBM films annealed at 160 °C. a) True‐color reflection POM image, acquired using slightly uncrossed polarizers (3° uncrossing angle) and a 50× objective lens. b) Magnified region of interest, cropped and expanded from the red box in (a). c–e) High magnification region of interest, cropped and expanded from the red box in (b), using different polarizer‐analyzer uncrossing angles: c) ‐3°, i.e., clockwise rotation of the analyzer; d) 0°, i.e., crossed polarizers; e) +3°, i.e., counter‐clockwise rotation of the analyzer. f–h) Falsely colored grayscale versions of (c–e). All regions of interest (c–h) share a common scale bar in (d). i–k) Pixel‐by‐pixel correlation analysis of the different grayscale images: i) 0° correlation with ‐3°; j) 0° correlation with +3°; k) +3° correlation with ‐3° images. The Pearson correlation coefficients are shown above each scatter plot.

The bright and dark features in POM did, however, exhibit optical activity with opposite handedness (**Figure** **5**).^[^ ^53^, ^54^, ^55^, ^56^, ^57^ ^]^ For an example film of regio‐regular P3HT:PCBM annealed at 160 °C (Figure 5a,b), we focused on one region of interest to compare different rotation directions of the analyzer relative to the polarizer (Figure 5c–e). The optical activity was observed as previously reported for chiral liquid crystals formed from achiral molecules^[^ ^53^, ^54^, ^55^, ^56^, ^57^ ^]^: under slightly uncrossed polarizers, with a clockwise rotation of the analyzer (i.e., ‐3° uncrossing angle), certain sub‐micron domains appeared bright compared with other domains (Figure 5c,f); while using a counter‐clockwise rotation of the analyzer (i.e., +3° uncrossing angle), the previously bright domains became dark, and the previously dark domains became bright (Figure 5e,h). In fact, the ‐3° and +3° POM images exhibited a very strong anti‐correlation, with a Pearson correlation coefficient of ‐0.90 (Figure 5k). Under crossed polarizers, the film exhibited very clear domain boundaries, likely separating domains with opposite handedness (Figure 5d,g).^[^ ^54^ ^]^ Since the film was not completely dark under crossed polarizers, there were still regions that exhibited birefringence, with the brighter regions attributed to regions of higher crystallinity compared to the darker regions. Some of the bright features in the cross polarized orientation correlated with the bright features in either the ±3° images (Figure 5i,j, positive slope), whereas others had anti‐correlation (Figure 5i,j, negative slope). This type of chiral behavior has been previously attributed to dark conglomerate phases of liquid crystals,^[^ ^53^, ^54^, ^55^, ^56^, ^57^ ^]^ which have similar behavior to lyotropic sponge phases.^[^ ^53^ ^]^ As conjugated polymers have been shown to exhibit lyotropic liquid crystalline states,^[^ ^58^, ^59^, ^60^ ^]^ these bright and dark domains are attributed primarily to the liquid crystalline nature of these organic semiconductor films.

Further sub‐microscopic correlative measurements of the local degree of crystallinity, molecular orientation, and chemical analysis, such as confocal Raman microscopy or AFM‐infrared nano‐spectroscopy, would be beneficial to fully confirm the origin of these different domains. We note additionally that rotation of the sample for a fixed angle between the polarizer and analyzer led to the highly crystalline PCBM needles appearing dark when parallel to the polarizer or analyzer, and bright when orthogonal (Figures S11 and S12, Supporting Information).

### 2.5 Reflection POM as a Rapid Diagnostic Tool for In‐Line Fabrication of Organic Optoelectronic Devices

While the results in this work have focused on P3HT:PCBM, we have also observed similar features from several other organic semiconductor systems so far, including PCDTBT,^[^ ^40^ ^]^ PCE‐10:ITIC, and PM6:Y6.^[^ ^61^ ^]^ We believe that combining reflection POM with an in‐line fabrication facility can help rapidly diagnose film non‐uniformities and compare degrees of crystallinity and phase segregation between films. The reflection geometry is perfectly suitable for automated characterization in a roll‐to‐roll production line, whereby active layers could be imaged immediately after deposition onto device stacks. Different processing conditions could be applied and rapidly characterized with the contrast from reflection POM being a figure of merit. Future work will continue investigating additional organic active layer materials, half device stacks, processing conditions, and to couple POM with an imaging spectroscopy setup.

## 3 Conclusion

In summary, we demonstrated that reflection POM can be used as a rapid, nondestructive tool to evaluate the crystallinity and phase segregation of organic semiconductor blend thin films. We outlined the optimal imaging conditions required to maximize contrast for POM images of organic semiconductor thin films, which we showed are using reflection POM with Köhler illumination and slightly uncrossed polarizers, having an uncrossing angle of ±3°. We quantitatively showed that the contrast in POM directly correlates with the degree of P3HT crystallinity within P3HT:PCBM films, and that the contrast increases even further for high degrees of phase segregation between P3HT and PCBM. We identified the origin of the bright and dark domains observed in POM as symmetry‐broken liquid crystalline phases, such as the dark conglomerate phase, and that they have no correlation with surface topography. Finally, we outlined how reflection POM could be integrated as a rapid diagnostic tool into in‐line organic optoelectronic device fabrication facilities. Future work in our lab will correlate POM images with confocal Raman microscopy maps to develop a better understanding of the origin of the enhanced contrast observed from POM.

## 4 Experimental Section

### Materials and Sample Preparation

Regio‐regular poly(3‐hexylthiophene‐2,5‐diyl) (P3HT) with average molecular weight of 50–75 kDa and [6,6]‐phenyl‐C_61_‐butyric acid methyl ester (PCBM) were purchased from Sigma Aldrich and 1‐Material, respectively. Anhydrous chlorobenzene was obtained from Sigma Aldrich and used as received. Solutions and thin films were prepared based on the protocol reported by Campoy‐Quiles et al.^[^ ^8^ ^]^ Chlorobenzene was used to dissolve P3HT and PCBM in a 1:1 weight ratio with a total concentration of 30 mg mL^−1^. Following, the solutions were stirred overnight at 40 °C. Glass substrates were cleaned sequentially in deionized water, acetone, and isopropanol for 15 min each while sonicating. The substrates also went through an oxygen plasma treatment for 15 min. The solution was then spin‐coated at 4000 rpm for 60 s, then annealed at different temperatures for 10 min. All sample preparation steps were processed inside a glovebox with N_2_ atmosphere.

### Polarized Optical Microscopy

A Nikon Eclipse LV100 POL optical microscope with a tungsten halogen lamp source was used for both bright‐field (BF) and POM imaging. The excitation path was polarized in‐plane, and a second polarizer (i.e., analyzer), was used to collect the light reflected from the samples. The analyzer was rotated for changing the polarizer‐analyzer uncrossing angle, with 0.1° precision. Films were imaged with either 20× (numerical aperture, NA = 0.45), 50× (NA = 0.80), or 100× (NA = 0.90) objectives; unless otherwise mentioned, the 50× objective was mostly used. For BF imaging, the analyzer was removed, but the polarizer was still used; there was no difference in the image contrast with or without the single polarizer. Brightness and contrast of the true‐color POM images were adjusted individually in Microsoft PowerPoint.

### Image Analysis

All image analysis was conducted using Python in Visual Studio Code, using Jupyter notebooks. Images were imported to Python and converted to grayscale (without any processing) using the Python Imaging Library (Pillow). The conversion applies a standard weighted sum to each of the red (R), green (G), and blue (B) pixel channels to reflect human perception, which is most sensitive to green and least to blue: Grayscale = 0.2989*R + 0.5870*G + 0.1140*B.^[^ ^62^ ^]^ From the grayscale images, histograms were generated by flattening the image arrays to 1D arrays. RMS contrast (*C*) was calculated using the following equation:

(1) $$C=\sqrt{\langle {\left(I_{\mathrm{roi}}-\langle I_{\mathrm{roi}}\rangle \right)}^{2}\rangle }$$

where *I*_roi_ is the pixel intensity array from the region of interest, and <*I*_roi_> is the mean pixel intensity from the region of interest.^[^ ^63^ ^]^

### Optical Spectroscopy

UV–VIS absorption spectra of films were recorded using a Lambda 950 UV/VIS/NIR spectrophotometer (PerkinElmer) coupled with an integrating sphere, in the transmission geometry. Absorbance values were baseline subtracted at a range where P3HT:PCBM has no absorption (i.e., 850 nm). Corrected PL spectra were obtained by using a Fluorolog modular spectrofluorometer (Horiba) with an excitation wavelength of 515 nm, an angle of incidence of 30°, excitation and emission slit widths of 4 nm, and acquisition time of 0.3 s per point. A 550 nm long pass filter was used to remove the excitation.

### Atomic Force Microscopy

Surface topography measurements were obtained using a Dimension Icon AFM (Veeco Instruments) in tapping (non‐contact) mode.

## Conflict of Interest

The authors declare no conflict of interest.

## Supporting information



Supporting Information

## Data Availability

The data that support the findings of this study are available from the corresponding author upon reasonable request.
