# Supplementary material for: On the Use of Reflection Polarized Optical Microscopy for Rapid Comparison of Crystallinity and Phase Segregation of P3HT:PCBM Thin Films
Source: Macromol Rapid Commun. 2024 Oct 21;45(24):2400577. doi: 10.1002/marc.202400577 (PMC11661667; doi:10.1002/marc.202400577)
Supplement: Supplementary file 1 — Supporting Information [file MARC-45-2400577-s001.pdf]

Macromolecular Rapid Communications

Supporting Information

for *Macromol. Rapid Commun.*, DOI 10.1002/marc.202400577

On the Use of Reflection Polarized Optical Microscopy for Rapid Comparison of Crystallinity and Phase Segregation of P3HT:PCBM Thin Films

Rawan A. Alzahrani, Nisreen Alshehri, Alaa A. Alessa, Doha A. Amer, Oleksandr Matiash, Catherine S. P. De Castro, Shahidul Alam, José P. Jurado, Julien Gorenflot, Frédéric Laquai and Christopher E. Petoukhoff**

Supporting Information
On the Use of Reflection Polarized Optical Microscopy for Rapid Comparison of Crystallinity and Phase Segregation of P3HT:PCBM Thin Films

Rawan A. Alzahrani,[1] Nisreen Alshehri,[1,2] Alaa A. Alessa,[1] Doha A. Amer,[1] Oleksandr Matiash,[1] Catherine S. P. De Castro,[1] Shahidul Alam,[1] José P. Jurado,[1] Julien Gorenflot,[1] Frédéric Laquai,[1,#,] and Christopher E. Petoukhoff[1,]**

1. King Abdullah University of Science and Technology (KAUST), Physical Sciences and Engineering Division (PSE), KAUST Solar Center (KSC), Thuwal, 23955-6900, Kingdom of Saudi Arabia
2. Physics and Astronomy Department, College of Sciences, King Saud University, Riyadh 12372, Kingdom of Saudi Arabia

[#]Present address for Prof. Dr. Frédéric Laquai: Physical Chemistry and Spectroscopy of Energy Materials, Department of Chemistry, Ludwig-Maximilians-Universität München, Butenandtstraße 5-13 (E), D-81377 München, Germany

*E-mail for correspondence: frederic.laquai@kaust.edu.sa, christopher.petoukhoff@kaust.edu.sa

Supporting Information Outline

1. Examples of POM Imaging of Organic Semiconductors 2
2. Reduction of Aberrations by Control of Aperture and Field Stops 4
3. Optimizing the Polarizer-Analyzer Uncrossing Angle for Highest Contrast 5
4. Quantifying P3HT Crystallinity by Spectral Deconvolution of Absorption 6
5. Estimating P3HT:PCBM Phase Segregation by Photoluminescence Spectroscopy 8
6. Additional Optical Microscopy Images of P3HT:PCBM Films 8
7. Correlative Analysis between AFM and POM 10
8. Details of Constructing Overlays between hi-res AFM and POM 11
9. Additional AFM-POM Correlations and Surface Roughness 15
10. Rotation of PCBM crystals in POM 16
11. References 18

1. Examples of POM Imaging of Organic Semiconductors

Table S1. Examples of POM imaging of organic semiconductors from literature. Examples include primarily conjugated polymers or conjugated small molecules, as well as several examples of organic liquid crystals. The analyzer-polarizer angle is listed as either crossed, for 90° between the two, or slightly uncrossed, for angles $\lesssim 90°$. R and T refer to reflection and transmission geometries. Note that the majority of studies do not report whether they employed R or T, but as T is generally more widely used, it can be assumed that unless specifically noted, T was likely used. The length scale refers to the scale bar value provided in the image. The "limitations with respect to this work" column highlights how our current study stands apart from these previous examples.

Organic Semiconductor Type	Analyzer-Polarizer angle	R or T	Length scale	Limitations with respect to this work	Reference
Identification of highly-oriented polymer chains					
uniaxial alignment of P3HT nanofibrils by solvent directional evaporation	crossed	?	100 μm	showed orientation direction only; aberrations likely present	Liu, … Han, Langmuir, 27, (2011) 4212.
oriented P3HT nanofibrils by inclining evaporation	crossed	?	200 μm	showed orientation direction only	Gao, …, Han, Chinese J. Poly. Sci., 31, (2013), 610.
highly ordered P3HT from self-assembly by capillary confinement	crossed	?	500 μm	showed orientation direction only	Kleinhenz, … Reichmanis, Chem. Mater., 27, (2015), 2687.
stretch-oriented P3HT on tape	crossed	R	1 mm	showed orientation direction only	Koch, … Stingelin, Prog. Poly. Sci., 38, (2013) 1978.
highly-oriented P3HT:PCBM films prepared via epitaxial crystallization	crossed	?	100 μm	showed orientation direction only	Memon, … Sun, J. Phys. Chem. B, 123, (2019) 7233.
uniaxially aligned films of thiophene polymers by capillary action	crossed (with phase compensator)	?	300 μm	showed orientation direction only	Higashi, … Ozaki, Appl. Phys. Express, 4, (2011) 091602.
Aligned F8T2 polymer by thermal treatment and rubbed polyimide	crossed	?	250 nm (error)	images too low resolution; scale clearly incorrect	Kinder, … Petroff, Synth. Met., 146, (2004) 181.
anisotropic supramolecular ordering by UV irradiation	crossed	?	100 μm	showed orientation direction only	Chang, … Reichmanis, Adv. Funct. Mater., 24, (2014) 4457.
Imaging of aggregated nanostructures					
P3HT nanowire arrays by deposition of high MW polymer on inclined substrate	crossed	?	100 μm	Images too dark to see any type of contrast from pristine films; minimal contrast from HM-P3HT	Li, … Luscombe, J. Mat. Chem. C, 5, (2017) 5128.

Nucleation and growth of P3HT spherulitic domains	crossed	?	50 μm	Very strong contrast, but from highly crystalline P3HT not practical for optoelectronic applications	Crossland, … Ludwigs, Adv. Funct. Mater., 21, (2021) 518.
Ordered P3HT aggregate nanostructures	crossed	?	50 μm	Good contrast from highly aggregated P3HT; not much from pristine film.	Neto, … Sampaio, Adv. Mater., 30, (2018) 1705052.
Crystalline aggregated of PDI molecule	crossed	?	20 μm	Could only see intensity from highly crystalline aggregates	Tintori, … Welch, Soft Matter, 15, (2019), 5138.
Imaging of dark conglomerate liquid crystal phases [Note: these are not semiconductors, but relevant for this work]					
Chiral isotropic LC from achiral molecules	slightly uncrossed	T	300 μm	Not an organic semiconductor.	Hough, ... Clark, Science, 325, (2009) 452.
Homochiral structure from achiral LC trimers	slightly uncrossed	T	200 μm	Not an organic semiconductor.	Yoshizawa, ... Yamamoto, J. Phys. Chem. B, 120, (2016) 4843.
Chiral nematic LC phase from achiral H-shaped LCs	slightly uncrossed	T	100 μm	Not an organic semiconductor.	Sayama and Yoshizawa, J. Mater. Chem. C, 7, (2019) 6905.
Dark conglomerate phase from chiral bent-core LCs	slightly uncrossed	?	100 μm	Not an organic semiconductor.	Baghla, ... Pal, J. Mater. Chem. C, 12, (2024) 3915.
Dark conglomerate phase from achiral bent core molecule	slightly uncrossed	?	500 μm	Not an organic semiconductor.	Kim, ... Choi, Polymers, 14, (2022) 2823.
Imaging of organic semiconductor thin films applicable for optoelectronic devices					
P3HT pristine thin films, varying concentration	crossed	?	100 μm	Some contrast observed, but images seem to have significant aberrations	Huang, … Chang, Macromol., 41, (2008) 7485.
P3HT:PCBM vary annealing temperature	?	?	50 nm (error)	Incorrect scale bar, non-uniform film thickness, no contrast	Motaung, … Cummings, J. Mater. Sci., 48, (2013) 1763.
Benzotrithiophene co-polymer thin films	crossed	R	20 μm	Showed enhanced contrast from lower MW polymer, but no quantitative analysis.	Schroeder, ... McCulloch, Chem. Mater., 23, (2011) 4025.
P3HT:PCBM thin films with different regio-regularity	slightly uncrossed	R	20 μm	Showed enhanced contrast with increasing crystallinity, but no quantitative analysis.	Alam, ... Laquai, J. Phys. Energy, 6, (2024) 025013.

2. Reduction of Aberrations by Control of Aperture and Field Stops

The role of the aperture stop is to reduce stray light from the source, enabling only paraxial rays from the light source to illuminate the sample. This helps to reduce aberrations that distort the POM images, preventing sharp images and decreasing the contrast. Figure S1a shows a series of POM images taken with slightly uncrossed polarizers of P3HT:PCBM annealed at 160 °C with various degrees of aperture stop closure. When the aperture stop was fully opened (i.e., 0% closed), severe aberrations blurred the sub-microscopic features, reducing the overall sharpness and contrast. When the aperture stop was partially closed (~70%), the aberrations were reduced and the contrast improved, but the features were still blurry. Only by closing the aperture stop to its minimum value (i.e., 100% closed) could the aberrations be reduced almost entirely, resulting in a sharp image with high contrast.

The field stop further helps to reduce unwanted stray light and diffraction effects produced by the aperture stop (Figure 1a and Figure S1b). Figure S1b shows a series of POM images taken from the same region as Figure S1a, in each case with the aperture stop fully closed, with varying degrees of field stop closure. By closing the field stop halfway (i.e., ~50%), higher contrast was achieved compared to the field stop being fully opened. As the field stop was closed more, the sharpness and contrast of the image continued to improve. However, it is important to note that since the field stop is in a conjugate image plane as the sample, closing the field stop also reduced the field of view of the full image. For our polarized optical microscope and CCD, closing the field stop more than ~50% resulted in the leaves of the stop appearing in the image.

We quantified improvements to the POM imaging conditions by comparing the RMS contrast for different degrees of aperture and field stop closure (Figure S1c). As the aperture stop closed, the RMS contrast increased from 5.44 when fully opened to 20.36 when closed to its minimum value (i.e., 100% closed). As the field stop additionally closed, the RMS contrast increased to 28.03 when closed 50% to its maximum value of 37.15 when closed ~90%. Practically, for most of the POM images in this work, we keep the aperture stop fully closed and the field stop 50% closed, in order to maximize both the contrast and the field of view.

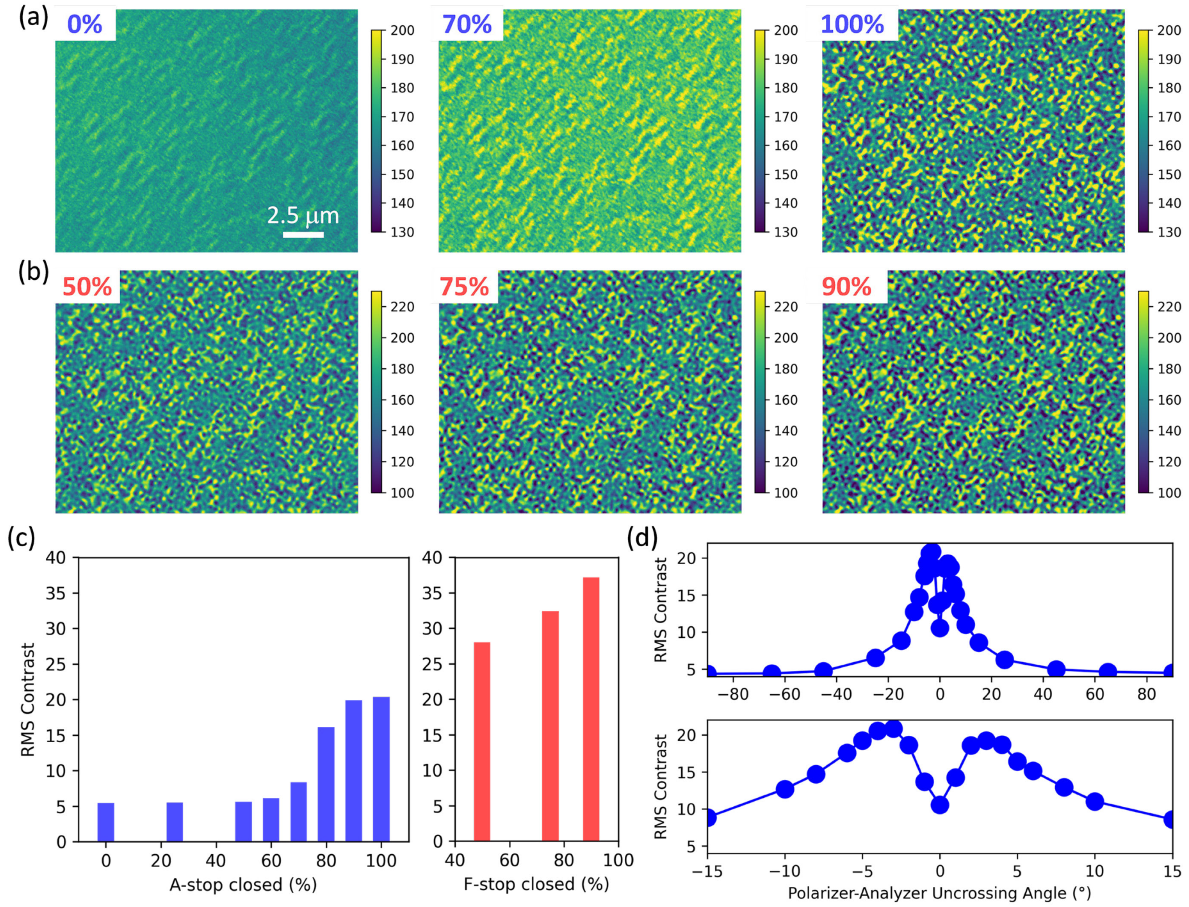

Figure S1. Methods of reducing aberrations and enhancing contrast for reflection polarized optical microscopy (POM). Grayscale POM regions of interest in (a) and (b) were taken from an identical region from a P3HT:PCBM film annealed at 160 °C using a 50× objective lens. All images were collected using slightly uncrossed polarizers, with an uncrossing angle of 3° (i.e., 87° between the polarizer and the analyzer). The scale bar for all images is shown in the first image within (a). (a) Images were acquired with various degrees of aperture (A-) stop closure. Note that these values were rough estimates from closing the A-stop lever, and that 0% closure is equivalent to the A-stop being fully opened, while 100% closure means the lever was closed to its maximum value (i.e., smallest diameter aperture), but light could still pass through. In these cases, the field (F-) stop was fully opened (i.e., 0% closure). (b) Images were acquired with various degrees of the F-stop closure. Note that these values were also rough estimates, and that all values greater than 50% led to a reduced overall field of view from the sample. In these cases, the A-stop was fully closed. (c) RMS contrast computed from POM images with varying degrees of A- and F-stop closure. (d) RMS contrast computed from POM images with varying the uncrossing angle between the polarizer and the analyzer. Here, the A-stop was fully closed, and the F-stop was 50% closed. Top: full angular range; bottom: magnified angular range, focused on the "slightly uncrossed" polarizers angles. Note that the maximum contrast was achieved for -3° uncrossing angle.

3. Optimizing the Polarizer-Analyzer Uncrossing Angle for Highest Contrast

We quantitatively compared the polarizer-analyzer uncrossing angle to determine the best angle for maximum contrast (Figure S1d). As demonstrated in Figure 2, the crossed polarized geometry did not give as high contrast as the slightly uncrossed configuration. Here, we further demonstrate that the contrast was maximized for an uncrossing angle of ±3°, with any uncrossing angle in the range of 2° ~ 6° having contrast greater than 15, which was more than 3 times larger than parallel polarizer configuration (i.e., uncrossing angle of ±90°). For most of

the POM images in this work, unless otherwise specified, we fix the uncrossing angle to be 3° (i.e., angle between the polarizer and analyzer to be 87°).

4. Quantifying P3HT Crystallinity by Spectral Deconvolution of Absorption

In order to quantify the degree of P3HT crystallinity for the P3HT:PCBM films annealed at different temperatures, we conducted a spectral deconvolution of the UV-VIS absorption spectra, following the methods described before by Spano,[1] Neher,[2] and Österbacka.[3] The absorption spectra were measured for each P3HT:PCBM film annealed at different temperatures (Figure S2a), and a reference spectrum for PCBM doped into polystyrene (PS) in a 50:50 ratio. The PCBM absorption was scaled for each film (Table S2) and subtracted from each of the P3HT:PCBM absorption spectra to determine the total P3HT absorption within each of the blend films (Figure S2b). Next, the crystalline (i.e., aggregate) region of each P3HT absorption spectra was fit with the following equation to account for the vibronic progression, based on absorption/emission from H-aggregates in the case of weak electronic coupling:[1, 2]

$$A(E) = A_0 \sum_{m=0} \left(\frac{S^m}{m!}\right) \times \left(1 - \frac{We^{-S}}{2E_p} \sum_{n \neq m} \frac{S^n}{n!\,(n-m)}\right)^2 \times \exp\left(-\frac{\left(E - E_{0-0} - mE_p - \frac{1}{2}WS^m e^{-S}\right)^2}{2\sigma^2}\right) \quad \text{(S1)}$$

where A is the absorbance as a function of photon energy (E), A_0 is a proportionality constant, S is the Huang-Rhys factor, m and n are different vibrational levels, W is the exciton bandwidth, E_p is the intermolecular vibrational energy, $E_{0\text{-}0}$ is the 0-0 transition energy, and σ is the Gaussian linewidth. Following the work of Turner and Neher,[2] the free parameters in the fit of the P3HT absorption spectra are: W, $E_{0\text{-}0}$, σ, and A_0; S is fixed at the value of 1.0, and E_p is taken to be 0.179 eV. The absorption spectra were fit within the range of 1.8 eV to 2.24 eV, where the aggregate region absorbs strongly (Figure S2c-f). After subtracting the aggregate absorption from the total P3HT absorption, the amorphous P3HT absorption was determined (Figure S2c-f). Finally, the degree of P3HT crystallinity was calculated using the following equation:

$$\text{Degree of P3HT crystallinity} = \frac{A_\mathrm{agg}}{A_t}, \quad \text{(S2)}$$

where A_agg is the integrated area of the aggregate absorbance and A_t is the integrated area of the total P3HT absorbance. The fitting results and degree of P3HT crystallinity are summarized in Table S2.

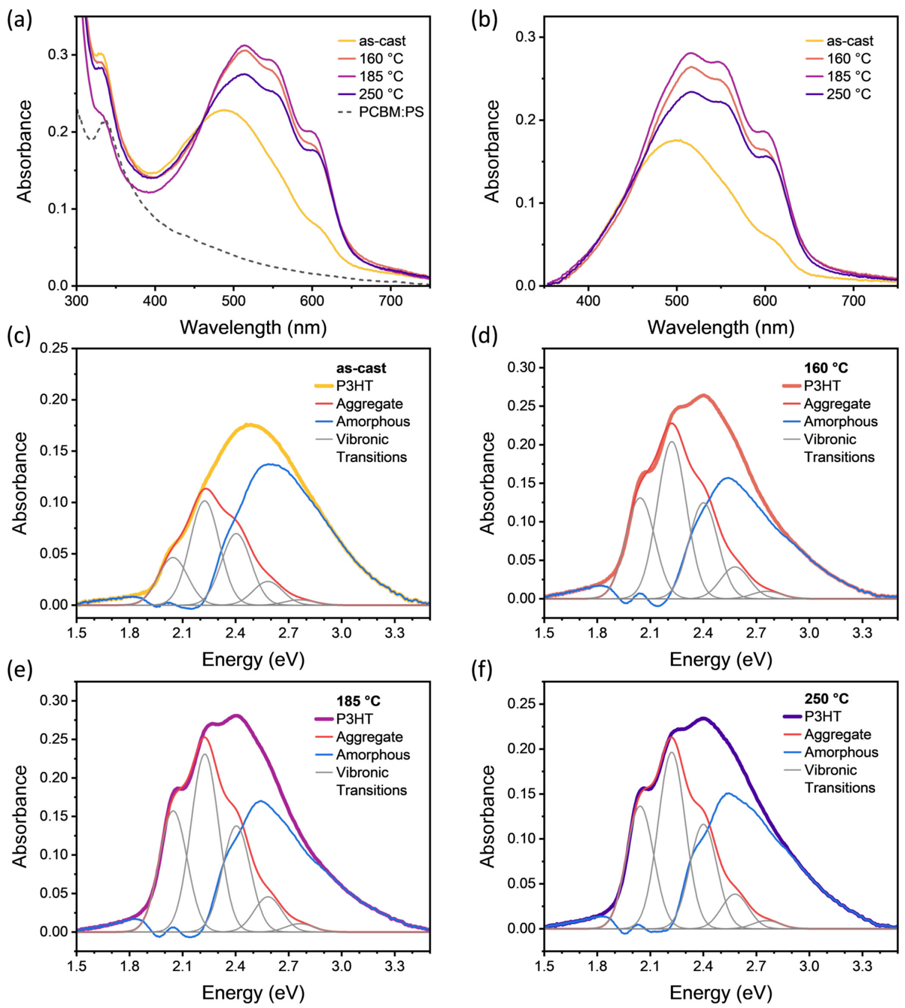

Figure S2. (a) UV-VIS absorption spectra of P3HT:PCBM films prepared with different thermal annealing conditions, and as-cast PCBM:PS reference film of similar thickness. (b) Contribution of P3HT absorption to that of the blend films, as determined by subtracting a scaled PCBM absorption spectrum from each of the spectra in (a). Note that the PCBM spectrum was scaled by different factors for each blend film (Table S2). (c-f) Spectral deconvolution of the aggregate absorbance from the total absorbance. The aggregate absorbance came from the best fit of the P3HT absorbance in the range of 1.8 eV to 2.24 eV using Equation S1. The amorphous P3HT absorbance component is the difference between the total measured P3HT absorbance and the fit of the aggregate absorbance. The individual vibronic transitions in the aggregate absorption are also shown.

Table S2. Results from UV-VIS spectral deconvolution and PL spectral integration, where T is temperature; f_{pcbm} is the proportionality constant to scale the PCBM:PS reference spectra for the subtraction in Figure S2b; W, $E_{0\text{-}0}$, σ, A_0 are the fitting parameters as described in this section, R^2 is the goodness-of-fit of the absorbance fitting; and Int. PL is the integrated photoluminescence spectral values, taken from the spectra in Figure S3.

T (°C)	f_{pcbm}	W (eV)	E_{0-0} (eV)	σ (eV)	A_0	% P3HT aggregates	R^2	Int. PL (x10^7)
as-cast	1.2	0.1986	2.0090	0.0835	0.0868	37.12	0.9849	5.337
160	1.2	0.1182	2.0209	0.0821	0.1856	52.96	0.9837	12.655
185	0.93	0.1031	2.0276	0.0784	0.2125	52.84	0.9881	13.152
250	1.13	0.0981	2.0242	0.0766	0.1815	50.65	0.9901	16.460

5. Estimating P3HT:PCBM Phase Segregation by Photoluminescence Spectroscopy

The degree of phase segregation between P3HT and PCBM can be traced by the photoluminescence (PL) intensity: higher intensity arises from greater phase segregation.[4-6] When blended with a strong electron acceptor, such as PCBM, the PL of P3HT becomes severely quenched due to good intermixing of P3HT with PCBM, arising from efficient exciton dissociation.[7, 8] For well-mixed, as-cast blends, typical domain sizes are comparable to the diffusion length of P3HT and PCBM excitons, which is ~5 nm to 10 nm.[7, 8] As phase segregation occurs by some external stressor, such as thermal annealing, the P3HT and PCBM domains become larger, leading to less efficient exciton dissociation and higher PL intensity. As such, relative changes in phase segregation can be traced by monitoring the integrated PL intensity. We observed that the PL intensity, and thus the degree of phase segregation, increased with increasing annealing temperature (Figure S3a and Table S2). As such, it was expected that for films annealed at 185 °C and 250 °C, the phase segregation was the highest, leading to formation of sub-microscale P3HT- and PCBM-rich domains (Figure S3b).

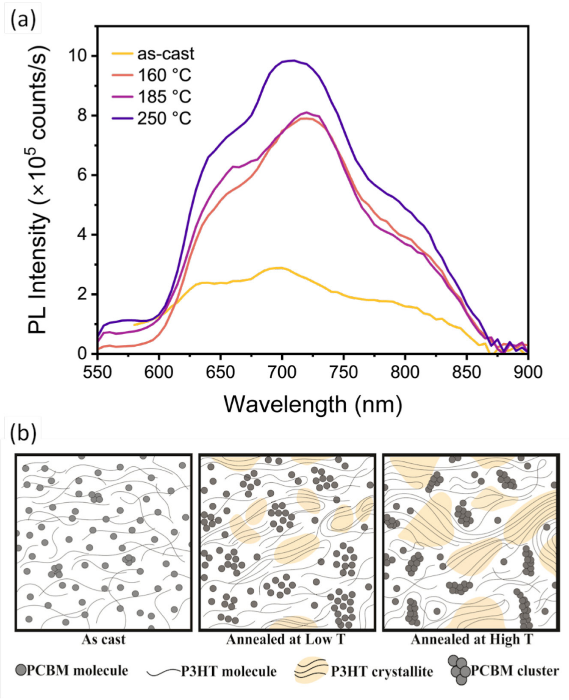

Figure S3. (a) Photoluminescence (PL) spectra of P3HT:PCBM films prepared with different thermal annealing conditions. (b) Schematic showing increase in P3HT crystallinity and phase segregation with increasing thermal annealing temperature.

6. Additional Optical Microscopy Images of P3HT:PCBM Films

Figure S4 shows bright-field (BF) and cross polarized POM images of the same 4 samples shown in Figure 4 of the main text. As discussed in the main text, the BF images lack contrast

to image sub-micron changes in the P3HT:PCBM mixed regions (Figure S4a-d). The cross polarized POM images did show changes in the background contrast (Figure S4e-h), but to a much lesser extent than the slightly uncrossed POM images shown in the main text (Figure 4).

Figure S5 shows optical microscopy images taken using a 100× objective, using different imaging conditions. The domain sizes are even easier to visualize at higher magnification. However, the objective lens was not polarization-corrected (unlike the other objective lenses used in all other images), and led to less uniform illumination, particularly in the cross polarized geometry.

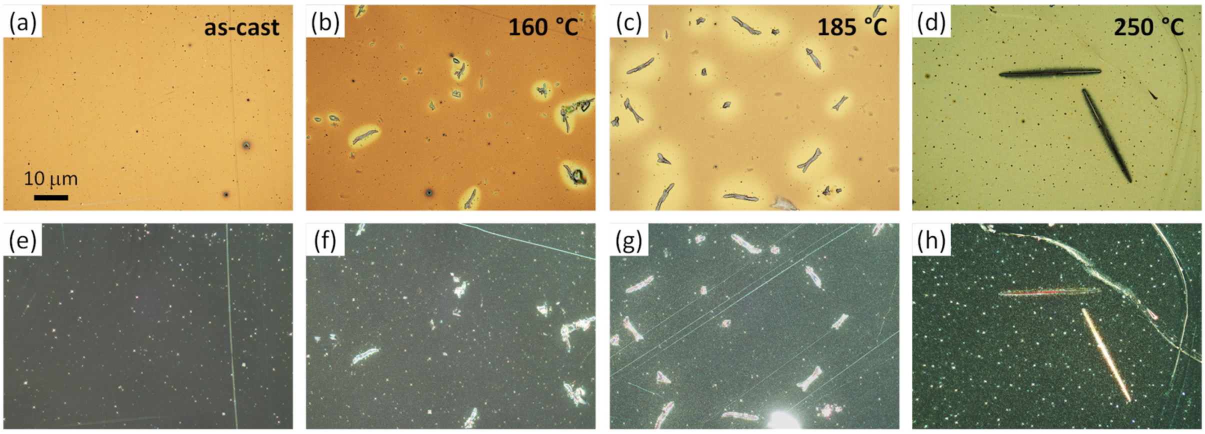

Figure S4. True-color optical microscopy images of P3HT:PCBM films annealed at different temperatures using (a-d) BF imaging and (e-h) cross polarized POM imaging. All images were acquired using a 50× objective lens, and the scale bar is shown in (a). The images were taken from identical regions as those shown in Figure 4a-d.

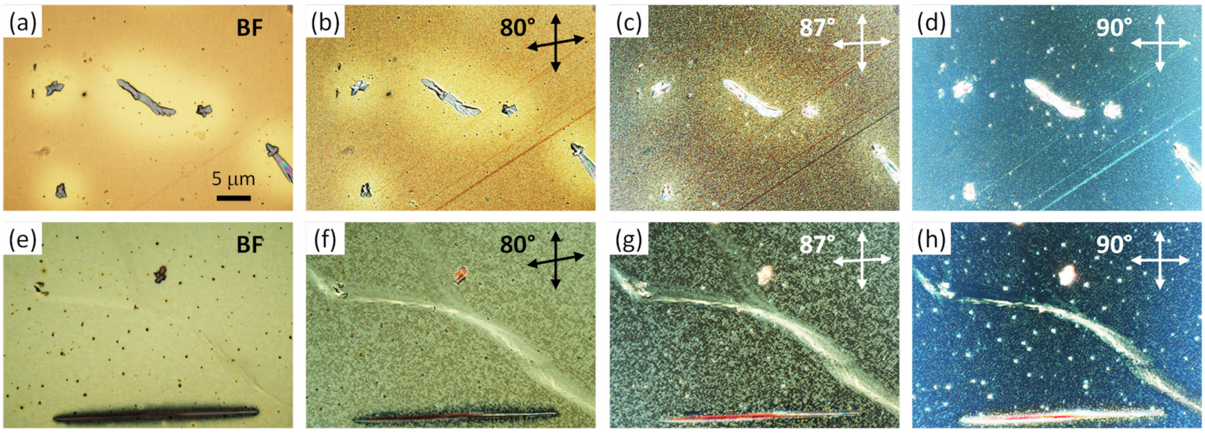

Figure S5. True-color optical microscopy images of P3HT:PCBM films annealed at 185 °C (a-d) and 250 °C (e-h) using different geometries: (a,e) BF; (b,f) POM with 10° uncrossing angle; (c,g) POM with 3° uncrossing angle; (d,h) POM with crossed polarizers. All images were acquired using a 100× objective lens, and the scale bar is shown in (a).

7. Correlative Analysis between AFM and POM

To prove that the intensity contrast in POM images originated from changes in the local degree of crystallinity or phase segregation, and not changes in surface topography, we correlated AFM surface topography measurements with identical regions imaged using POM from films annealed at 160 °C (Figure S6). The low-resolution AFM and POM images (Figure S6 a and b, respectively) show that we collected measurements at identical areas, as confirmed by the PCBM crystals, which act as reference markers for this correlation. To obtain a pixel-by-pixel overlay of the high-resolution AFM image with the POM image, we employed a differential evolution genetic algorithm to optimize the overlay of the low-resolution image onto the POM image (Figure S7). Conducting a pixel-by-pixel correlation of the low-resolution AFM with the POM image showed a modest correlation (Figure S6c), with a Pearson correlation coefficient of 0.52, demonstrating that the low-resolution AFM was effectively overlaid onto the POM image. We next scaled and shifted the high-resolution AFM image using known scaling factors (Figure S8) to overlay it onto the low-resolution AFM-image, ensuring we had pixel-by-pixel matching of the identical regions of interest with POM (Figure S6d,e and Figure S8). Conducting the pixel-by-pixel correlation showed that there was no correlation between the high-resolution AFM image and the POM image (Figure S6f), with a Pearson correlation coefficient of 0.02. This proves quantitatively that there were no correlations between the POM contrast and AFM surface topography.

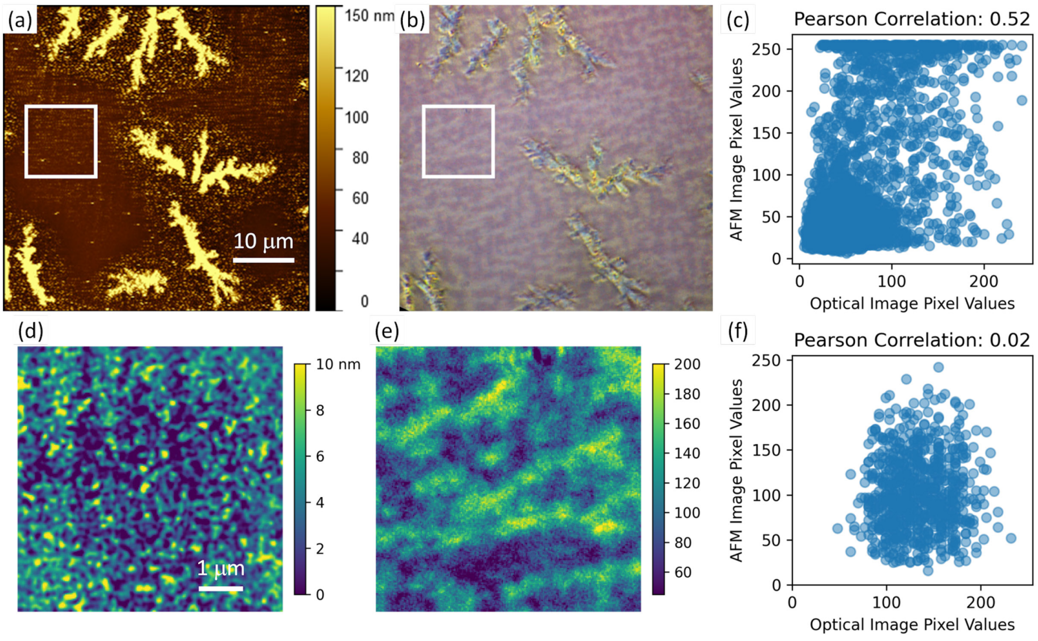

Figure S6. Correlative imaging of surface topography using AFM (a, d) and degree of crystallinity using POM (b, e). (a, b) Low magnification images, showing the region of interest for the comparisons. The POM image in (b) was acquired using a 100× objective and 3° uncrossing angle. (c) Pixel-by-pixel correlation

analysis of low magnification AFM vs POM images. The genetic algorithm-optimized correlation coefficient of 0.52 shows there was a moderate correlation between the 2 images, arising from the PCBM clusters. (d) High resolution AFM scan from the region of interest highlighted by the white box in panel (a). (e) Same image as in (b), magnified and cropped to the region of interest highlighted by the white box. (f) Pixel-by-pixel correlation analysis of high magnification AFM vs POM images. The correlation coefficient of 0.02 shows there was no correlation between the 2 images.

8. Details of Constructing Overlays between hi-res AFM and POM

To determine if surface topography was the origin of the intensity variation in the POM images, we conducted a pixel-by-pixel correlation analysis of our AFM and POM images, as introduced in the previous section. To make the overlays, we followed the following procedure, shown schematically in Figures S7 and S8:

Rough overlay of low-resolution AFM and POM:

1. We took a low-resolution AFM image (50 μm × 50 μm scan) in the same region as the POM image (Figure S7a). The region of interest contained several PCBM crystals as reference markers for making overlays.
2. The POM image, initially at 1500 pixels × 1500 pixels (from the cropped region of interest), was resized to match the pixel density of the AFM image (i.e., 853 pixels × 853 pixels).
3. The AFM images were converted to grayscale and made semi-transparent.
4. The AFM image was transformed by iterating through the following:
    a. x- and y- scaling
    b. rotation
    c. x- and y- translations
5. The transformed AFM image was then overlaid onto the POM image (Figure S7b).

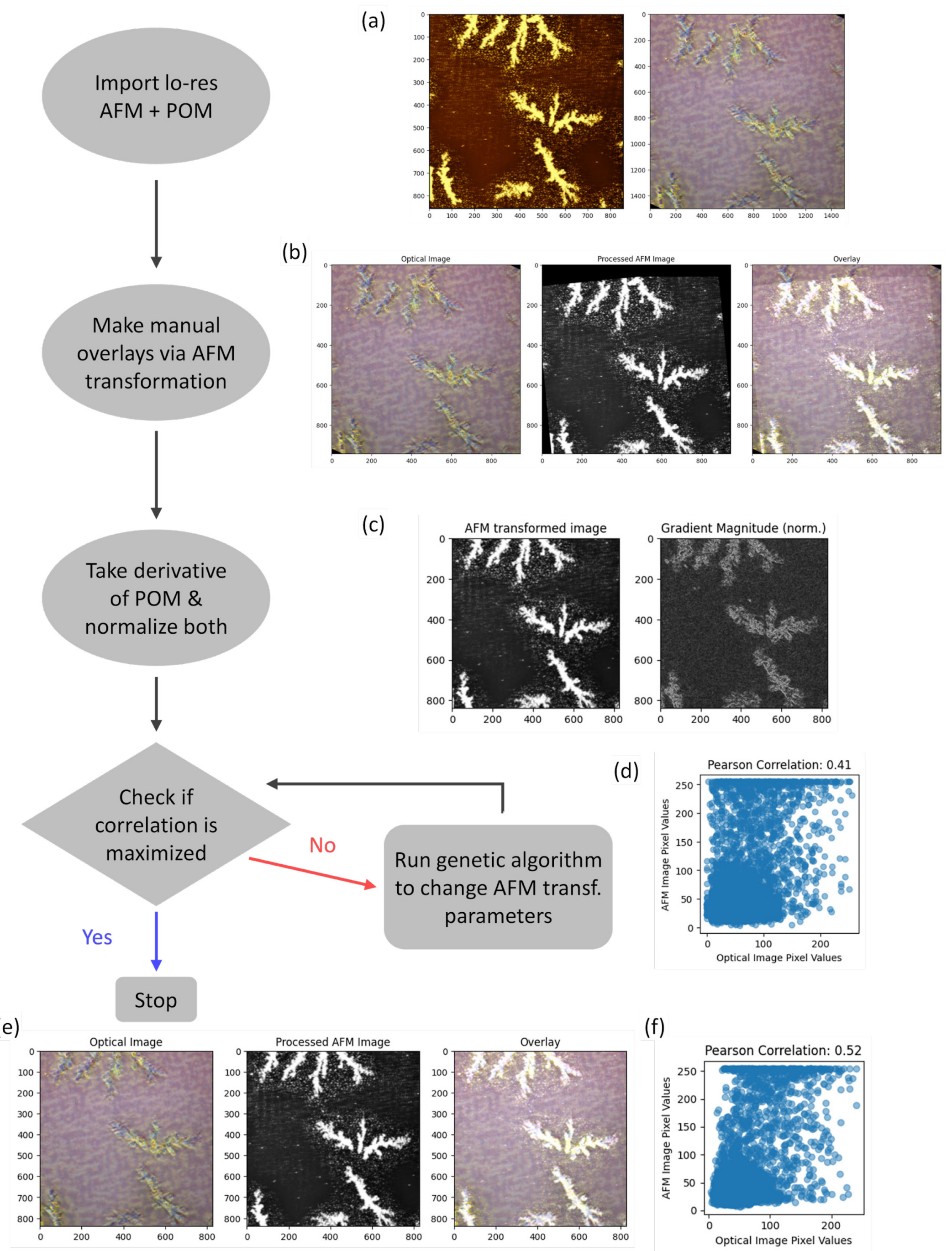

Figure S7. Procedure used to make overlays of low magnification AFM and POM images. Left: schematic representation of main steps involved. (a) Low magnification AFM and POM images taken from identical regions. (b) Manual overlay between POM and AFM, where the AFM image has been scaled, rotated, and translated to match the POM image. (c) Gradient of POM image plotted next to the AFM image, both normalized from 0 to 255 pixel intensity. (d) Pixel-by-pixel correlation analysis of the transformed AFM image and the gradient of POM image. (e) Final overlay between POM and AFM, after optimizing the AFM transformation parameters to maximize the correlation coefficient using a differential evolution genetic algorithm. (f) Pixel-by-pixel correlation analysis of the final transformed AFM image and the gradient of the POM image.

This procedure resulted in overlaying the identical regions for the low-resolution AFM and POM images, to the best estimate by eye. To improve the quality of the overlays, we employed

a differential evolution genetic algorithm to optimize the AFM transformation parameters, using the following procedure:

Improved overlay using genetic algorithm:

6. The POM image was converted to grayscale, and the gradient of the image was acquired. This accounted for the intensity variations in the PCBM clusters, which were not uniformly bright (Figure S7c).
7. The gradient of the POM image and the transformed AFM image were both normalized to have their pixel distributions span from 0 to 255.
8. The normalized images were flattened into 1D arrays.
9. The Pearson correlation coefficient was calculated from the 1D arrays (Figure S7d).
10. Using the AFM transformation parameters as fitting parameters and the Pearson correlation coefficient as the target, the transformation routine (Step 4) was input into a differential evolution genetic algorithm to iterate through the parameters until the correlation coefficient was maximized (Figure S7e).
11. The correlations were visualized by creating a scatter plot of the final 1D arrays (Figure S7f).

By doing this, we improved our confidence in the overlaid AFM and POM images. The correlation coefficient and resulting scatter plot also demonstrate the accuracy of these methods by comparing images with very obvious features.

Precise overlay of high-resolution AFM and POM:

We next took a high-resolution AFM scan (15 μm × 15 μm) from a region of interest (Figure S8a) and applied a known transformation to this image (i.e., x- and y- offsets) to overlay it onto the original low-resolution AFM image. Since both AFM images were acquired with 256 pixels × 256 pixels, we resized the low-resolution AFM image to have a proportionally larger number of pixels (i.e., 50 μm / 15 μm × 256 pixels = 853 pixels) in order to stitch the low-resolution AFM into the high-resolution AFM image without loss of pixel resolution from the high-resolution image (Figure S8b).

Finally, we applied our genetic algorithm-optimized AFM transformation parameters to the combined high- + low-resolution AFM image to overlay all 3 images: POM with low- and high-

resolution AFM scans (Figure S8c). This ensures we have as high confidence as possible in creating our high-resolution AFM – POM overlays.

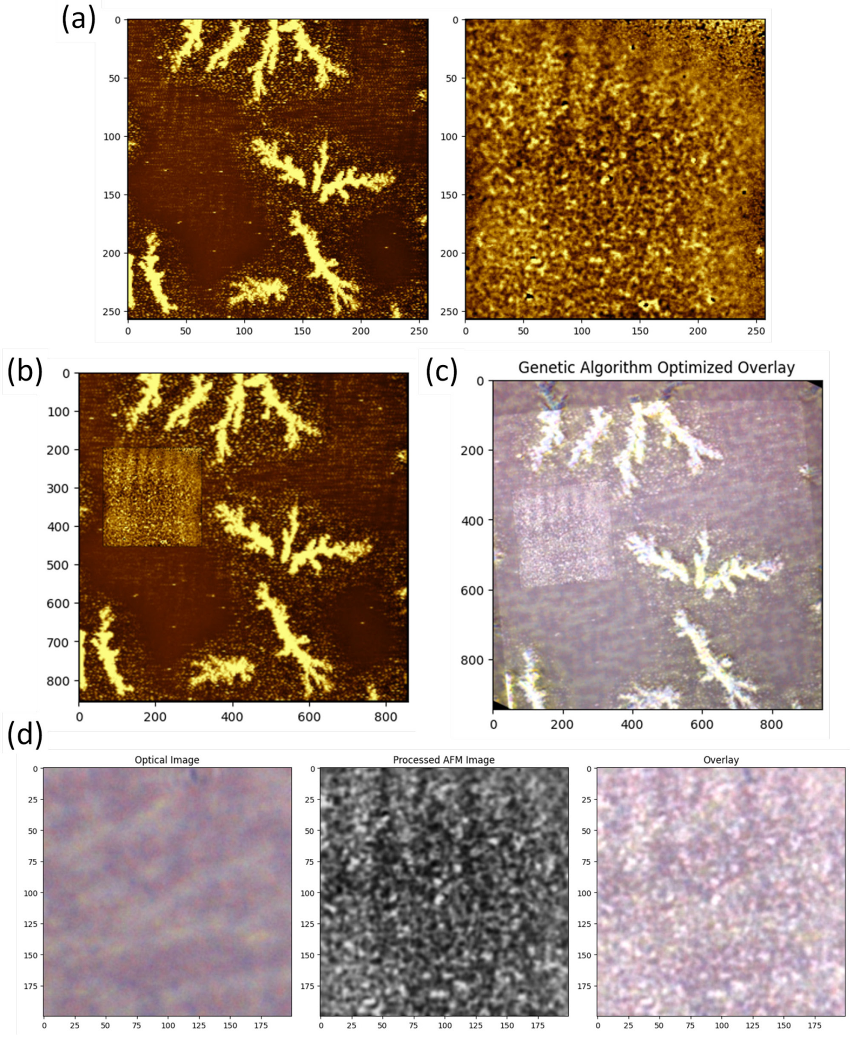

Figure S8. Procedure used to make overlays of high magnification AFM and POM images. (a) Original low magnification (left) and high magnification (right) AFM images. (b) Manual overlay between low and high magnification AFM images, using known offsets and scan sizes for scaling and translating. (c) Overlay between low and high magnification AFM and POM images, using the previously optimized transformation parameters for the AFM images. (d) Cropped region of interest containing the high-resolution AFM data and the POM data from identical regions. These images were then converted to grayscale and falsely colored for Figure S6.

We then cropped each image to the identical region of interest (Figure S8d), converted the high-resolution AFM and POM regions of interest to grayscale, calculated the Pearson correlation coefficient, and created the scatter plot of flattened images (Figure S6d-f). This proves quantitatively that there were no correlations between the POM contrast and AFM surface topography.

WILEY-VCH

9. Additional AFM-POM Correlations and Surface Roughness

This lack of correlation was qualitatively confirmed with 4 separate regions, none of which showed a visual correlation between the surface topography and the intensity variations measured using POM (Figure S9). However, while there were no correlations between the surface topography and intensity variations from POM images, there was generally an increase in the RMS surface roughness of the P3HT:PCBM films with increasing annealing temperature, up to 185 °C (Figure S10). The film annealed at 250 °C had RMS roughness less than the 160 °C sample but greater than the as-cast sample, further confirming the trend in degree of P3HT crystallinity as reported based on the spectroscopy results.

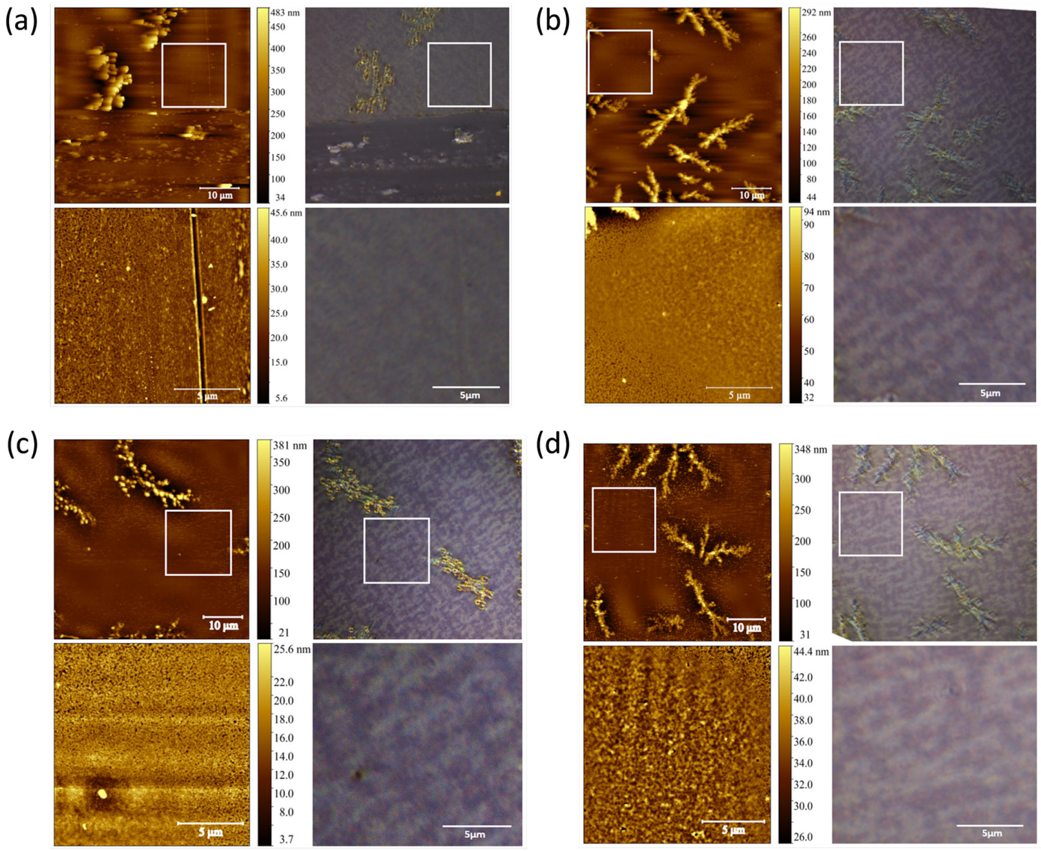

Figure S9. Qualitative correlative imaging of surface topography using AFM (left columns) and degree of crystallinity using POM (right columns) for 4 different regions. The POM images were acquired using a 100× objective and 3° uncrossing angle. The magnified POM images were cropped from the original images.

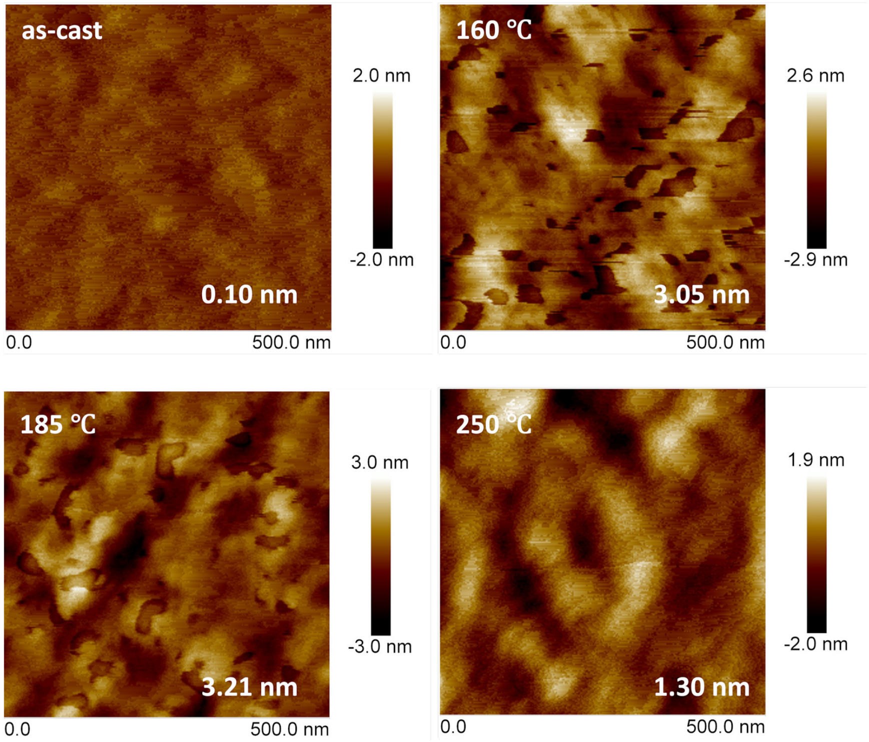

Figure S10. AFM surface roughness measurements for P3HT:PCBM films annealed at different temperatures. The RMS surface roughness values are displayed on the bottom right of each image.

10. Rotation of PCBM crystals in POM

By varying the rotation of the sample with respect to the polarizer and analyzer, the intensity of PCBM crystallites changes from dark to bright (Figures S11, S12). This effect can be observed either with slightly off-cross polarizers (i.e., 3° uncrossing angle; Figure S11) or with fully crossed-polarizers (Figure S12). Imaging the films at a random sample angle always led to some PCBM crystals appearing dark, and others appearing very bright (Figure S11a). PCBM crystals aligned parallel to either the polarizer or the analyzer appeared darkest (e.g., crystal 1 in Figure S11b), whereas those orthogonal to the polarizer and analyzer appeared brightest (e.g., crystal 2 in Figure S11b). As the sample was rotated, the intensity of the crystals changed to become dark whenever aligned with the polarizers (e.g., crystal 1 in Figure S11b or e; crystal 2 in Figure S11c, f) and to become brightest whenever orthogonal to the polarization direction (e.g., crystal 2 in Figure S11d, e; crystal 4 in Figure S11b, c, e, and f). This shows the high quality of the needle-like PCBM micro crystals that emerge from extended phase segregation.

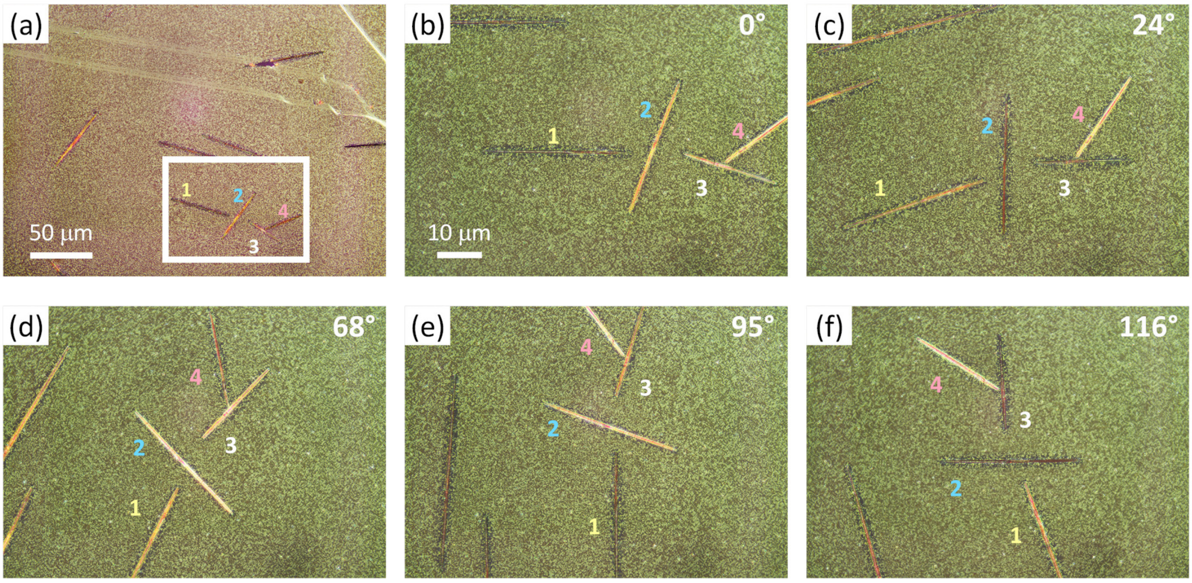

Figure S11. POM images of the P3HT:PCBM films annealed at 250 °C at different sample rotation angles. (a) low magnification (20×) image, showing the region of interest. (b-f) high magnification (50×) images acquired with different rotation angles of the sample stage (scale bar shown in (b)). All images were acquired with the same conditions, and with 3° polarizer-analyzer uncrossing angle. Four selected crystals were identified in (a) and labeled for each high magnification image at different rotation angles.

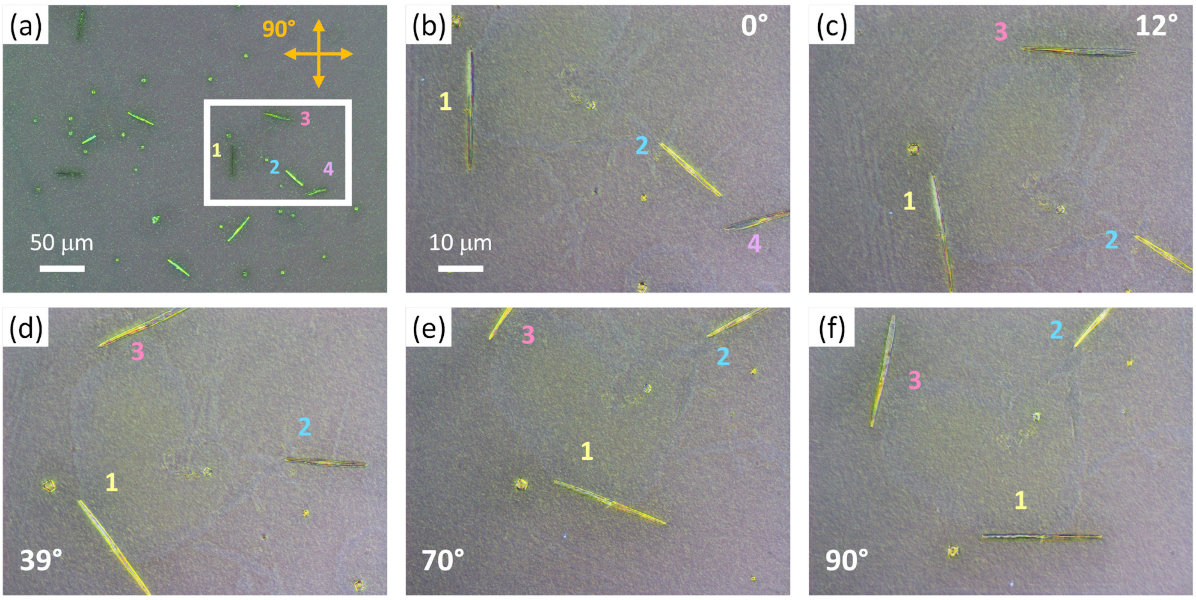

Figure S12. POM images of a P3HT:PCBM film annealed at 250 °C at different sample rotation angles. (a) low magnification (20×) image, showing the region of interest. (b-f) high magnification (50×) images acquired with different rotation angles of the sample stage (scale bar shown in (b)). All images were acquired with the same conditions, and with crossed polarizers. Four selected crystals were identified in (a) and labeled for each high magnification image at different rotation angles.

11. References

[1] J. Clark, C. Silva, R. H. Friend, F. C. Spano, *Phys Rev Lett* **2007**, *98*, 206406.
[2] S. T. Turner, P. Pingel, R. Steyrleuthner, E. J. W. Crossland, S. Ludwigs, D. Neher, *Advanced Functional Materials* **2011**, *21*, 4640.
[3] S. Wilken, D. Scheunemann, S. Dahlström, M. Nyman, J. Parisi, R. Österbacka, *Advanced Electronic Materials* **2021**, *7*, 2001056.
[4] D. Spoltore, T. Vangerven, P. Verstappen, F. Piersimoni, S. Bertho, K. Vandewal, N. Van den Brande, M. Defour, B. Van Mele, A. De Sio, J. Parisi, L. Lutsen, D. Vanderzande, W. Maes, J. V. Manca, *Organic Electronics* **2015**, *21*, 160.
[5] W. C. Tsoi, S. J. Spencer, L. Yang, A. M. Ballantyne, P. G. Nicholson, A. Turnbull, A. G. Shard, C. E. Murphy, D. D. C. Bradley, J. Nelson, J.-S. Kim, *Macromolecules* **2011**, *44*, 2944.
[6] S. S. van Bavel, M. Bärenklau, G. de With, H. Hoppe, J. Loos, *Advanced Functional Materials* **2010**, *20*, 1458.
[7] U. Zhokhavets, T. Erb, H. Hoppe, G. Gobsch, N. Serdar Sariciftci, *Thin Solid Films* **2006**, *496*, 679.
[8] A. J. Pearson, T. Wang, R. A. L. Jones, D. G. Lidzey, P. A. Staniec, P. E. Hopkinson, A. M. Donald, *Macromolecules* **2012**, *45*, 1499.
